# Supplementary material for: Characterization of a hemolytic and antibiotic-resistant Pseudomonas aeruginosa strain S3 pathogenic to fish isolated from Mahananda River in India
Source: PLoS One. 2024 Mar 28;19(3):e0300134. doi: 10.1371/journal.pone.0300134 (PMC10977779; doi:10.1371/journal.pone.0300134)
Supplement: S1 File — (DOCX) [file pone.0300134.s001.docx]

**Supporting Information**

**Characterization of a hemolytic and antibiotic-resistant *Pseudomonas aeruginosa* strain S3 pathogenic to fish isolated from Mahananda River in India**

**Dipanwita Ghosh^1^, Preeti Mangar^2^, Abhinandan Choudhury^1^, Anoop Kumar^1^, Aniruddha Saha^2^, Protip Basu^3^, Dipanwita Saha^1*^**

^1^Department of Biotechnology, University of North Bengal, Siliguri, West Bengal, India.

^2^Department of Botany, University of North Bengal, Siliguri, West Bengal, India.

^3^Department of Botany, Siliguri College, West Bengal, India.

*** Correspondence:**
E-mail - [dipanwitasaha@nbu.ac.in](mailto:dipanwitasaha@nbu.ac.in) (DS)

**S1 Table. Screening of the hemolytic isolates for the presence of extracellular virulence factors**

| **Strains** | **Virulence factors** | | | | | |
| --- | --- | --- | --- | --- | --- | --- |
|  | **Haemolysin** | **Protease** | **Lipase** | **DNase** | **Siderophore** | **Amylase** |
| **S3** | + | + | + | + | + | - |
| **A1** | + | - | + | + | - | - |
| **J1** | + | - | - | + | - | - |
| **P6** | + | - | - | + | - | - |
| **S5** | + | + | + | + | - | - |
| **S4** | + | + | - | + | - | + |
| **B3** | + | - | - | + | - | + |
| **M14** | + | + | - | + | - | + |
| **M2** | + | + | - | + | - | - |
| **A3** | + | - | + | + | - | + |
| **B2** | + | - | - | + | - | - |
| **C2** | + | + | - | + | - | - |
| **C4** | + | - | - | + | - | - |
|  |  |  |  |  |  |  |
| **Strains** | **Hemolysin** | **Protease** | **Lipase** | **DNase** | **Siderophore** | **Amylase** |
| **D1** | + | - | - | + | + | + |
| **P8** | + | + | - | + | - | - |
| **B6** | + | + | - | + | - | + |
| **P14** | + | + | - | + | - | + |
| **B8** | + | - | + | + | - | - |
| **M3** | + | - | + | + | - | - |
| **MH5** | + | - | + | + | + | - |
| **MH7** | + | + | - | + | - | - |
| **MH8** | + | - | - | - | + | + |
| **F1** | + | - | + | - | - | - |
| **F2** | + | + | - | + | - | + |
| **F9** | + | + | - | - | - | - |
| **F10** | + | + | - | + | - | - |

**‘+’ indicates positive**

**‘-’ indicates negative**

**S2 Table.** Prediction of genes related to virulence factors of *Pseudomonas aeruginosa* S3 according to VFDB database.

| **Gene** | **Product** | **Source ID** | **Source Organism** |
| --- | --- | --- | --- |
| *alg44* | Alginate polymerisation protein Alg44, membrane fusion protein | VFG000124 | *P.aeruginosa* PAO1 |
| *pscL* | Type III secretion cytoplasmic protein (YscL) | VFG000220 | *P.aeruginosa* PAO1 |
| *pscI* | Type III secretion cytoplasmic protein (YscI) | VFG000217 | *P.aeruginosa* PAO1 |
| *flhB* | Flagellar biosynthesis protein FlhB | VFG001262 | *P.aeruginosa* PAO1 |
| *hsiB1/vipA* | T6SS component TssB (ImpB/VipA) | VFG002069 | *P.aeruginosa* PAO1 |
| *flgJ* | Flagellar protein FlgJ [peptidoglycan hydrolase] | VFG001243 | *P.aeruginosa* PAO1 |
| *pchB* | Isochorismate pyruvate-lyase (EC 4.2.99.21) [pyochelin] siderophore @ Isochorismate pyruvate-lyase (EC 4.2.99.21) of siderophore biosynthesis | VFG000170 | *P.aeruginosa* PAO1 |
| *xcpU* | General secretion pathway protein H | VFG000179 | *P.aeruginosa* PAO1 |
| *pilI* | type IV pili signal transduction protein PilI | VFG001227 | *P.aeruginosa* PAO1 |
| *exoY* | Adenylate cyclase ExoY (EC 4.6.1.1) | VFG000150 | *P.aeruginosa* PAO1 |
| *pscG* | Type III secretion spans bacterial envelope protein (YscG) | VFG000215 | *P.aeruginosa* PAO1 |
| *fliC* | Flagellin protein FlaB | VFG001246 | *P.aeruginosa* PAO1 |
| *algD* | GDP-mannose 6-dehydrogenase (EC 1.1.1.132) | VFG000122 | *P.aeruginosa* PAO1 |
| *xcpV* | General secretion pathway protein I | VFG000178 | *P.aeruginosa* PAO1 |
| *ptxR* | Transcriptional regulator PtxR, LysR family | VFG044083 | *P.aeruginosa* PAO1 |
| *mucA* | Sigma factor RpoE negative regulatory protein RseA | VFG000134 | *P.aeruginosa* PAO1 |
| *pcrR* | Type III secretion low calcium response protein (LcrR) | VFG000199 | *P.aeruginosa* PAO1 |
| *fpvA* | Outer membrane ferripyoverdine receptor FpvA, TonB-dependent @ Ferric siderophore receptor, TonB dependent | VFG000163 | *P.aeruginosa* PAO1 |
| *algJ* | Alginate O-acetyltransferase AlgJ, inner membrane | VFG000131 | *P.aeruginosa* PAO1 |
| *flgH* | Flagellar L-ring protein FlgH | VFG001241 | *P.aeruginosa* PAO1 |
| *mucP* | Intramembrane protease RasP/YluC, implicated in cell division based on FtsL cleavage | VFG015000 | *P.aeruginosa* PAO1 |
| *rhlI* | N-butanoyl-L-homoserine lactone synthase @ N-acyl-L-homoserine lactone synthase, LuxI family (EC 2.3.1.184) | VFG000153 | *P.aeruginosa* PAO1 |
| *algL* | Alginate lyase AlgL (EC 4.2.2.3) | VFG000129 | *P.aeruginosa* PAO1 |
| *tags* | ABC-type antimicrobial peptide transport system, permease component | VFG002058 | *P.aeruginosa* PAO1 |
| *pvcA* | PvcA protein, related to known isonitrile synthases | VFG044079 | *P.aeruginosa* PAO1 |
| *pvdM* | Putative dipeptidase, pyoverdin biosynthesis PvdM | VFG015983 | *P.aeruginosa* PAO1 |
| *algZ* | Alginate biosynthesis two-component system sensor histidine kinase AlgZ/FimS | VFG000120 | *P.aeruginosa* PAO1 |
| *algK* | Alginate export system AlgK/AlgE, periplasmic component AlgK | VFG000125 | *P.aeruginosa* PAO1 |
| *aprA* | Secreted alkaline metalloproteinase (EC 3.4.24.-), PrtA/B/C/G homolog | VFG000144 | *P.aeruginosa* PAO1 |
| *pvcB* | PvcB protein, related to amino acid oxidizing enzymes | VFG044080 | *P.aeruginosa* PAO1 |
| *tse2* | hypothetical protein | VFG041046 | *P.aeruginosa* PAO1 |
| *pvdO* | PvdO, pyoverdine responsive serine/threonine kinase (predicted by OlgaV) | VFG016005 | *P.aeruginosa* PAO1 |
| *fleP* | Two-component system sensor histidine kinase | VFG014343 | *P.aeruginosa* PAO1 |
| *xcpZ* | General secretion pathway protein M | VFG000174 | *P.aeruginosa* PAO1 |
| *pilR* | Type IV fimbriae expression regulatory protein PilR | VFG001214 | *P.aeruginosa* PAO1 |
| *fliL* | Flagellar basal body-associated protein FliL | VFG014486 | *P.aeruginosa* PAO1 |
| *pvdD* | Pyoverdine sidechain non-ribosomal peptide synthetase PvdD @ Siderophore biosynthesis non-ribosomal peptide synthetase modules | VFG000161 | *P.aeruginosa* PAO1 |
| *pilF* | Type IV pilus biogenesis protein PilF | VFG001217 | *P.aeruginosa* PAO1 |
| *exsD* | Type III secretion negative regulator (LscZ) | VFG000209 | *P.aeruginosa* PAO1 |
| *pscR* | Type III secretion inner membrane protein (YscR,SpaR,HrcR,EscR,homologous to flagellar export components) | VFG000188 | *P.aeruginosa* PAO1 |
| *pilJ* | twitching motility protein PilJ | VFG001228 | *P.aeruginosa* PAO1 |
| *fimU* | type 4 fimbrial biogenesis protein FimU | VFG001216 | *P.aeruginosa* PAO1 |
| *pilK* | Chemotaxis protein methyltransferase CheR (EC 2.1.1.80) | VFG001229 | *P.aeruginosa* PAO1 |
| *tagF/pppB* | T6SS associated component TagF (ImpM) | VFG002062 | *P.aeruginosa* PAO1 |
| *fimT* | Type IV fimbrial biogenesis protein FimT | VFG001215 | *P.aeruginosa* PAO1 |
| *pvdS* | Sigma factor PvdS, controlingpyoverdin biosynthesis | VFG000162 | *P.aeruginosa* PAO1 |
| *algE* | Alginate export system Algk/AlgE, outer membrane porin AlgE | VFG000126 | *P.aeruginosa* PAO1 |
| *pcrG* | Type III secretion cytoplasmic plug protein (LcrG) | VFG000200 | *P.aeruginosa* PAO1 |
| *clpV1* | T6SS AAA+ chaperone ClpV (TssH) | VFG002076 | *P.aeruginosa* PAO1 |
| *algP/algR3* | Alginate regulatory protein AlgP, positive transcriptional regulator of AlgD | VFG000117 | *P.aeruginosa* PAO1 |
| *pvdA* | L-ornithine 5-monooxygenase (EC 1.13.12.-), PvdA of pyoverdin biosynthesis @ Siderophore biosynthesis protein, monooxygenase | VFG000159 | *P.aeruginosa* PAO1 |
| *fliE* | Flagellar hook-basal body complex protein FliE | VFG001250 | *P.aeruginosa* PAO1 |
| *pilT* | Twitching motility protein PilT | VFG001223 | *P.aeruginosa* PAO1 |
| *waaG* | UDP-glucose:(heptosyl) LPS alpha1,3-glucosyltransferase WaaG | VFG000139 | *P.aeruginosa* PAO1 |
| *pvdF* | Pyoverdine synthetase PvdF, N5-hydroxyornithine formyltransferase | VFG016016 | *P.aeruginosa* PAO1 |
| *phzM* | Phenazine-specific methyltransferase PhzM | VFG000172 | *P.aeruginosa* PAO1 |
| *flgL* | Flagellar hook-associated protein FlgL | VFG001245 | *P.aeruginosa* PAO1 |
| *fleS* | Flagellar sensor histidine kinase FleS | VFG014369 | *P.aeruginosa* PAO1 |
| *Wzy* | Oligosaccharide repeat unit polymerase Wzy | VFG000138 | *P.aeruginosa* PAO1 |
| *plcH* | Phospholipase C (EC 3.1.4.3) =>hemolyticPlcH | VFG000157 | *P.aeruginosa* PAO1 |
| *pcr3* | Type III secretion protein SctX | VFG000196 | *P.aeruginosa* PAO1 |
| *exsB* | Type III secretion transporter lipoprotein (YscW,VirG) | VFG000207 | *P.aeruginosa* PAO1 |
| *fliS* | Flagellar biosynthesis protein FliS | VFG014330 | *P.aeruginosa* PAO1 |
| *hsiC1/vipB* | T6SS component TssC (ImpC/VipB) | VFG002070 | *P.aeruginosa* PAO1 |
| *xcpX* | General secretion pathway protein K | VFG000176 | *P.aeruginosa* PAO1 |
| *pilW* | Type IV fimbrial biogenesis protein PilW | VFG001208 | *P.aeruginosa* PAO1 |
| *pilX* | Type IV fimbrial biogenesis protein PilX | VFG001209 | *P.aeruginosa* PAO1 |
| *fliH* | Flagellar assembly protein FliH | VFG001253 | *P.aeruginosa* PAO1 |
| *pchE* | Dihydroaeruginoate synthetase PchE, non-ribosomal peptide synthetase modules @ Siderophore biosynthesis non-ribosomal peptide synthetase modules | VFG000166 | *P.aeruginosa* PAO1 |
| *flhA* | Flagellar biosynthesis protein FlhA | VFG001263 | *P.aeruginosa* PAO1 |
| *waaP* | Lipopolysaccharide core heptose(I) kinase RfaP | VFG000140 | *P.aeruginosa* PAO1 |
| *rhlA* | RhlA, 3-(3-hydroxyalkanoyloxy)alkanoic acids (HAAs) synthase | VFG000156 | *P.aeruginosa* PAO1 |
| *fliR* | Flagellar biosynthesis protein FliR | VFG001261 | *P.aeruginosa* PAO1 |
| *pvdE* | PvdE, pyoverdine ABC export system, fused ATPase and permease components | VFG000160 | *P.aeruginosa* PAO1 |
| *mbtH-like* | MbtH-like NRPS chaperone | VFG044088 | *P.aeruginosa* PAO1 |
| *pscD* | Type III secretion inner membrane protein (YscD,homologous to flagellar export components) | VFG000212 | *P.aeruginosa* PAO1 |
| *xcpS* | General secretion pathway protein F | VFG000181 | *P.aeruginosa* PAO1 |
| *algG* | Poly (beta-D-mannuronate) C5 epimerase AlgG (EC 5.1.3.-) | VFG000127 | *P.aeruginosa* PAO1 |
| *fleQ* | Flagellar regulatory protein FleQ | VFG001248 | *P.aeruginosa* PAO1 |
| *algR* | Alginate biosynthesis two-component system response regulator AlgR | VFG000119 | *P.aeruginosa* PAO1 |
| *pscP* | Type III secretion protein (YscP) | VFG000190 | *P.aeruginosa* PAO1 |
| *xcpT* | General secretion pathway protein G | VFG000180 | *P.aeruginosa* PAO1 |
| *flgI* | Flagellar P-ring protein FlgI | VFG001242 | *P.aeruginosa* PAO1 |
| *alg8* | Alginate polymerase/glycosyltransferase Alg8 | VFG000123 | *P.aeruginosa* PAO1 |
| *motD* | Flagellar motor rotation protein MotB | VFG014720 | *P.aeruginosa* PAO1 |
| *hsiJ1* | T6SS component TssK (ImpJ/VasE) | VFG002065 | *P.aeruginosa* PAO1 |
| *motB* | Flagellar motor rotation protein MotB | VFG014681 | *P.aeruginosa* PAO1 |
| *mucB* | Sigma factor RpoE negative regulatory protein RseB precursor | VFG000135 | *P.aeruginosa* PAO1 |
| *pcrH* | Type III secretion chaperone protein for YopD (SycD) | VFG000202 | *P.aeruginosa* PAO1 |
| *motY* | Putative outer membrane protein | VFG014733 | *P.aeruginosa* PAO1 |
| *xcpA/pilD* | Leader peptidase (Prepilin peptidase) (EC 3.4.23.43) / N-methyltransferase (EC 2.1.1.-) | VFG000114 | *P.aeruginosa* PAO1 |
| *ppkA* | T6SS Serine/threonine protein kinase (EC 2.7.11.1) PpkA | VFG002060 | *P.aeruginosa* PAO1 |
| *exsC* | Unknown, probably involved in type III secretion | VFG000205 | *P.aeruginosa* PAO1 |
| *hsiH1* | T6SS component TssG (ImpH/VasB) | VFG002075 | *P.aeruginosa* PAO1 |
| *chpA* | still frameshift probable component of chemotactic signal transduction system | VFG001231 | *P.aeruginosa* PAO1 |
| *flgE* | Flagellar hook protein FlgE | VFG001238 | *P.aeruginosa* PAO1 |
| *tagR* | hypothetical protein | VFG002057 | *P.aeruginosa* PAO1 |
| *flgC* | Flagellar basal-body rod protein FlgC | VFG001236 | *P.aeruginosa* PAO1 |
| *pile* | Type IV pilus biogenesis protein PilE | VFG001212 | *P.aeruginosa* PAO1 |
| *tse3* | Bacterial lysozyme Tse3, effector of type VI secretion system | VFG041047 | *P.aeruginosa* PAO1 |
| *vgrG1a* | VgrG protein | VFG002077 | *P.aeruginosa* PAO1 |
| *fimV* | Probable type IV pilus assembly FimV-related transmembrane protein | VFG001230 | *P.aeruginosa* PAO1 |
| *toxA* | hypothetical protein | VFG000115 | *P.aeruginosa* PAO1 |
| *motA* | Flagellar motor rotation protein MotA | VFG014694 | *P.aeruginosa* PAO1 |
| *pilY1* | Type IV fimbrial biogenesis protein PilY1 | VFG001210 | *P.aeruginosa* PAO1 |
| *chpD* | Transcriptional regulator, AraC family | VFG001234 | *P.aeruginosa* PAO1 |
| *rhlB* | RhlB, TDP-rhamnosyltransferase 1 (EC 2.4.1.-) | VFG000155 | *P.aeruginosa* PAO1 |
| *fha1* | T6SS forkhead associated domain protein ImpI/VasC | VFG002067 | *P.aeruginosa* PAO1 |
| *popN* | Type III secretion outermembrane contact sensing protein (YopN,Yop4b,LcrE) | VFG000193 | *P.aeruginosa* PAO1 |
| *pilA* | Type IV pilin PilA | VFG000111 | *P.aeruginosa* PAO1 |
| *pilU* | Type IV pilus assembly ATPase component PilU | VFG001224 | *P.aeruginosa* PAO1 |
| *icmF1/tssM1* | T6SS component TssM (IcmF/VasK) | VFG002063 | *P.aeruginosa* PAO1 |
| *algU* | RNA polymerase sigma factor RpoE | VFG000121 | *P.aeruginosa* PAO1 |
| *phzS* | FAD-dependent monooxygenase PhzS | VFG000173 | *P.aeruginosa* PAO1 |
| *pvdQ* | Acyl-homoserine lactone acylase PvdQ (EC 3.5.1.-), quorum-quenching | VFG015950 | *P.aeruginosa* PAO1 |
| *tagQ* | Autotransporter adhesin | VFG041010 | *P.aeruginosa* PAO1 |
| *mucD* | HtrA protease/chaperone protein | VFG014950 | *P.aeruginosa* PAO1 |
| *fptA* | Outer membrane receptor for ferric-pyochelin FptA @ Outer membrane receptor for ferric siderophore | VFG000164 | *P.aeruginosa* PAO1 |
| *flgK* | Flagellar hook-associated protein FlgK | VFG001244 | *P.aeruginosa* PAO1 |
| *lip1* | T6SS secretion lipoprotein TssJ (VasD) | VFG002066 | *P.aeruginosa* PAO1 |
| *pvcC* | 4-hydroxyphenylacetate 3-monooxygenase (EC 1.14.14.9) | VFG044081 | *P.aeruginosa* PAO1 |
| *algI* | Probable poly(beta-D-mannuronate) O-acetylase (EC 2.3.1.-) | VFG000130 | *P.aeruginosa* PAO1 |
| *exsE* | FIG00962212: hypothetical protein | VFG000206 | *P.aeruginosa* PAO1 |
| *pilY2* | Type IV fimbrial biogenesis protein PilY2 | VFG001211 | *P.aeruginosa* PAO1 |
| *tagT* | ABC transporter, ATP-binding protein | VFG002059 | *P.aeruginosa* PAO1 |
| *pscN* | Type III secretion cytoplasmic ATP synthase (EC 3.6.3.14, YscN,SpaL,MxiB,HrcN,EscN) | VFG000192 | *P.aeruginosa* PAO1 |
| *pvdG* | ThioesterasePvdG involved in non-ribosomal peptide biosynthesis | VFG016069 | *P.aeruginosa* PAO1 |
| *pvdJ* | Pyoverdine sidechain non-ribosomal peptide synthetase PvdJ @ Siderophore biosynthesis non-ribosomal peptide synthetase modules | VFG016038 | *P.aeruginosa* PAO1 |
| *pscE* | Type III secretion protein (YscE) | VFG000213 | *P.aeruginosa* PAO1 |
| *pchA* | Isochorismate synthase (EC 5.4.4.2) @ Isochorismate synthase (EC 5.4.4.2) [pyochelin] siderophore @ Isochorismate synthase (EC 5.4.4.2) of siderophore biosynthesis | VFG000171 | *P.aeruginosa* PAO1 |
| *chpE* | probable chemotaxis protein | VFG001235 | *P.aeruginosa* PAO1 |
| *pchR* | Transcriptional regulator PchR | VFG000167 | *P.aeruginosa* PAO1 |
| *pilQ* | Type IV pilus biogenesis protein PilQ | VFG001218 | *P.aeruginosa* PAO1 |
| *pscH* | Type III secretion effector protein (YopR, encoded by YscH) | VFG000216 | *P.aeruginosa* PAO1 |
| *fliD* | Flagellar cap protein FliD | VFG001247 | *P.aeruginosa* PAO1 |
| *hsiF1* | T6SS lysozyme-like component TssE | VFG002073 | *P.aeruginosa* PAO1 |
| *exsA* | Type III secretion thermoregulatory protein (LcrF,VirF,transcription regulation of virulence plasmid) | VFG000208 | *P.aeruginosa* PAO1 |
| *xcpY* | General secretion pathway protein L | VFG000175 | *P.aeruginosa* PAO1 |
| *fliO* | Flagellar biosynthesis protein FliO | VFG001258 | *P.aeruginosa* PAO1 |
| *pvdI* | Pyoverdine sidechain non-ribosomal peptide synthetase PvdI @ Siderophore biosynthesis non-ribosomal peptide synthetase modules | VFG016041 | *P.aeruginosa* PAO1 |
| *pscB* | Type III secretion chaperone protein for YopN (SycN,YscB) | VFG000210 | *P.aeruginosa* PAO1 |
| *flip* | Flagellar biosynthesis protein FliP | VFG001259 | *P.aeruginosa* PAO1 |
| *xcpQ* | General secretion pathway protein D | VFG000184 | *P.aeruginosa* PAO1 |
| *waaC* | Lipopolysaccharide core heptosyltransferase I | VFG000142 | *P.aeruginosa* PAO1 |
| *pscU* | Type III secretion inner membrane protein (YscU,SpaS,EscU,HrcU,SsaU, homologous to flagellar export components) | VFG000185 | *P.aeruginosa* PAO1 |
| *flhF* | Flagellar biosynthesis protein FlhF | VFG001264 | *P.aeruginosa* PAO1 |
| *pilN* | Type IV pilus biogenesis protein PilN | VFG001221 | *P.aeruginosa* PAO1 |
| *fliG* | Flagellar motor switch protein FliG | VFG001252 | *P.aeruginosa* PAO1 |
| *xcpR* | General secretion pathway protein E | VFG000182 | *P.aeruginosa* PAO1 |
| *fliJ* | Flagellar protein FliJ | VFG001255 | *P.aeruginosa* PAO1 |
| *pilV* | Type IV fimbrial biogenesis protein PilV | VFG001207 | *P.aeruginosa* PAO1 |
| *fleR* | Flagellar two-component response regulator FleR | VFG001249 | *P.aeruginosa* PAO1 |
| *pscO* | Type III secretion spans bacterial envelope protein (YscO) | VFG000191 | *P.aeruginosa* PAO1 |
| *exoS* | putative exoenzyme T | VFG000147 | *P.aeruginosa* PAO1 |
| *pvdP* | Pyoverdine biosynthesis related protein PvdP | VFG015973 | *P.aeruginosa* PAO1 |
| *popB* | Type III secretion host injection protein (YopB) | VFG000203 | *P.aeruginosa* PAO1 |
| *fliI* | Flagellum-specific ATP synthase FliI | VFG001254 | *P.aeruginosa* PAO1 |
| *phzH* | Phenazine modifying protein PhzH / Asparagine synthetase [glutamine-hydrolyzing] (EC 6.3.5.4) | VFG015551 | *P.aeruginosa* PAO1 |
| *mucC* | Sigma factor RpoE regulatory protein RseC | VFG000136 | *P.aeruginosa* PAO1 |
| *fliK* | Flagellar hook-length control protein FliK | VFG014473 | *P.aeruginosa* PAO1 |
| *pcr2* | Type III secretion chaperone SycN | VFG000195 | *P.aeruginosa* PAO1 |
| *pilO* | Type IV pilus biogenesis protein PilO | VFG001220 | *P.aeruginosa* PAO1 |
| *pilG* | twitching motility protein PilG | VFG001225 | *P.aeruginosa* PAO1 |
| *waaF* | ADP-heptose--lipooligosaccharideheptosyltransferase II | VFG000143 | *P.aeruginosa* PAO1 |
| *exoT* | hypothetical protein | VFG000148 | *P.aeruginosa* PAO1 |
| *pchC* | Pyochelin biosynthetic protein PchC, predicted thioesterase @ Thioesterase in siderophore biosynthesis gene cluster | VFG000169 | *P.aeruginosa* PAO1 |
| *flgN* | Flagellar biosynthesis protein FlgN | VFG014668 | *P.aeruginosa* PAO1 |
| *mucE* | hypothetical protein | VFG014997 | *P.aeruginosa* PAO1 |
| *pilH* | twitching motility protein PilH | VFG001226 | *P.aeruginosa* PAO1 |
| *fliN* | Flagellar motor switch protein FliN | VFG001257 | *P.aeruginosa* PAO1 |
| *hsiA1* | T6SS component TssA (ImpA) | VFG002068 | *P.aeruginosa* PAO1 |
| *lasA* | LasA protease precursor | VFG000145 | *P.aeruginosa* PAO1 |
| *pchG* | Pyochelin biosynthetic protein PchG, oxidoreductase (NAD-binding) @ Thiazolinyl imide reductase in siderophore biosynthesis gene cluster | VFG001268 | *P.aeruginosa* PAO1 |
| *fliQ* | Flagellar biosynthesis protein FliQ | VFG001260 | *P.aeruginosa* PAO1 |
| *algB* | Alginate biosynthesis two-component system response regulator AlgB | VFG000116 | *P.aeruginosa* PAO1 |
| *alga* | Mannose-1-phosphate guanylyltransferase (EC 2.7.7.13) / Mannose-6-phosphate isomerase (EC 5.3.1.8) | VFG000133 | *P.aeruginosa* PAO1 |
| *waaA* | 3-deoxy-D-manno-octulosonic acid transferase (EC 2.4.99.12)(EC 2.4.99.13) | VFG000141 | *P.aeruginosa* PAO1 |
| *flgD* | Flagellar basal-body rod modification protein FlgD | VFG001237 | *P.aeruginosa* PAO1 |
| *fleI/flag* | Flagellar protein FlaG | VFG014304 | *P.aeruginosa* PAO1 |
| *pvcD* | hypothetical protein | VFG044082 | *P.aeruginosa* PAO1 |
| *chpC* | probable chemotaxis protein | VFG001233 | *P.aeruginosa* PAO1 |
| *hsiE1* | T6SS associated component TagJ (ImpE) | VFG002072 | *P.aeruginosa* PAO1 |
| *tse1* | hypothetical protein | VFG041030 | *P.aeruginosa* PAO1 |
| *pscS* | Type III secretion inner membrane protein (YscS,homologous to flagellar export components) | VFG000187 | *P.aeruginosa* PAO1 |
| *algW* | Outer membrane stress sensor protease DegS | VFG014984 | *P.aeruginosa* PAO1 |
| *pscT* | Type III secretion inner membrane protein (YscT,HrcT,SpaR,EscT,EpaR1,homologous to flagellar export components) | VFG000186 | *P.aeruginosa* PAO1 |
| *lasB* | Vibriolysin, extracellular zinc protease (EC 3.4.24.25) @ Pseudolysin, extracellular zinc protease (EC 3.4.24.26) | VFG000146 | *P.aeruginosa* PAO1 |
| *xcpP* | General secretion pathway protein C | VFG000183 | *P.aeruginosa* PAO1 |
| *algQ* | Alginate regulatory protein AlgQ, positive transcriptional regulator of AlgD | VFG000118 | *P.aeruginosa* PAO1 |
| *algX* | Alginate O-acetyltransferase AlgX, periplasmic | VFG000128 | *P.aeruginosa* PAO1 |
| *vgrG1b* | VgrG protein | VFG041011 | *P.aeruginosa* PAO1 |
| *pchH* | ABC efflux pump, fused inner membrane and ATPase subunits in pyochelin gene cluster @ Putative ABC iron siderophore transporter, fused permease and ATPase domains | VFG001267 | *P.aeruginosa* PAO1 |
| *popD* | Type III secretion host injection and negative regulator protein (YopD) | VFG000204 | *P.aeruginosa* PAO1 |
| *pcrD* | Type III secretion inner membrane channel protein (LcrD,HrcV,EscV,SsaV) | VFG000198 | *P.aeruginosa* PAO1 |
| *fliA* | RNA polymerase sigma factor for flagellar operon | VFG014629 | *P.aeruginosa* PAO1 |
| *vgrG1a* | VgrG protein | VFG002077 | *P.aeruginosa* PAO1 |
| *pvdL* | Pyoverdine chromophore precursor synthetase PvdL @ Siderophore biosynthesis non-ribosomal peptide synthetase modules | VFG016058 | *P.aeruginosa* PAO1 |
| *pcrV* | Type III secretion cytoplasmic LcrG inhibitor (LcrV,secretion and targeting control protein, V antigen) | VFG000201 | *P.aeruginosa* PAO1 |
| *pilC* | Type IV fimbrial assembly protein PilC | VFG000113 | *P.aeruginosa* PAO1 |
| *pilS* | Two-component sensor PilS | VFG001213 | *P.aeruginosa* PAO1 |
| *pilM* | Type IV pilus biogenesis protein PilM | VFG001222 | *P.aeruginosa* PAO1 |
| *pchI* | ABC efflux pump, fused inner membrane and ATPase subunits in pyochelin gene cluster @ Putative ABC iron siderophore transporter, fused permease and ATPase domains | VFG001266 | *P.aeruginosa* PAO1 |
| *hsiG1* | T6SS component TssF (ImpG/VasA) | VFG002074 | *P.aeruginosa* PAO1 |
| *pscK* | Type III secretion cytoplasmic protein (YscK) | VFG000219 | *P.aeruginosa* PAO1 |
| *pchD* | 2,3-dihydroxybenzoate-AMP ligase (EC 2.7.7.58) [pyochelin] siderophore @ 2,3-dihydroxybenzoate-AMP ligase (EC 2.7.7.58) of siderophore biosynthesis | VFG000168 | *P.aeruginosa* PAO1 |
| *flgF* | Flagellar basal-body rod protein FlgF | VFG001239 | *P.aeruginosa* PAO1 |
| *pscF* | Type III secretion cytoplasmic protein (YscF) | VFG000214 | *P.aeruginosa* PAO1 |
| *pvdH* | Pyoverdin biosynthesis protein PvdH, L-2,4-diaminobutyrate:2-oxoglutarate aminotransferase (EC 2.6.1.76) | VFG016046 | *P.aeruginosa* PAO1 |
| *Wzz* | hypothetical protein | VFG000137 | *P.aeruginosa* PAO1 |
| *pilB* | Type IV fimbrial assembly, ATPase PilB | VFG000112 | *P.aeruginosa* PAO1 |
| *algC* | Phosphoglucomutase (EC 5.4.2.2) @ Phosphomannomutase (EC 5.4.2.8) | VFG014893 | *P.aeruginosa* PAO1 |
| *chpB* | Chemotaxis response regulator protein-glutamate methylesteraseCheB (EC 3.1.1.61) | VFG001232 | *P.aeruginosa* PAO1 |
| *pcr1* | Type III secretion outermembrane negative regulator of secretion (TyeA) | VFG000194 | *P.aeruginosa* PAO1 |
| *flgA* | Flagellar basal-body P-ring formation protein FlgA | VFG014642 | *P.aeruginosa* PAO1 |
| *fliM* | Flagellar motor switch protein FliM | VFG001256 | *P.aeruginosa* PAO1 |
| *lasI* | N-(3-oxododecanoyl)-L-homoserine lactone synthase @ N-acyl-L-homoserine lactone synthase, LuxI family (EC 2.3.1.184) | VFG000154 | *P.aeruginosa* PAO1 |
| *dotU1* | T6SS outer membrane component TssL (ImpK/VasF) / OmpA/MotB domain | VFG002064 | *P.aeruginosa* PAO1 |
| *pvdN* | Pyoverdin biosynthesis protein PvdN, putative aminotransferase, class V | VFG015994 | *P.aeruginosa* PAO1 |
| *flgM* | Negative regulator of flagellin synthesis FlgM (anti-sigma28) | VFG014655 | *P.aeruginosa* PAO1 |
| *pscQ* | Type III secretion inner membrane protein (YscQ,homologous to flagellar export components) | VFG000189 | *P.aeruginosa* PAO1 |
| *fleN* | Flagellar synthesis regulator FleN | VFG001265 | *P.aeruginosa* PAO1 |
| *flgG* | Flagellar basal-body rod protein FlgG | VFG001240 | *P.aeruginosa* PAO1 |
| *hcp1* | T6SS component Hcp | VFG002071 | *P.aeruginosa* PAO1 |
| *pscC* | Type III secretion outermembrane pore forming protein (YscC,MxiD,HrcC, InvG) | VFG000211 | *P.aeruginosa* PAO1 |
| *xcpW* | General secretion pathway protein J | VFG000177 | *P.aeruginosa* PAO1 |
| *pchF* | Pyochelin synthetase PchF, non-ribosomal peptide synthetase module @ Siderophore biosynthesis non-ribosomal peptide synthetase modules | VFG000165 | *P.aeruginosa* PAO1 |
| *fliF* | Flagellar M-ring protein FliF | VFG001251 | *P.aeruginosa* PAO1 |
| *rhlC* | RhlC, TDP-rhamnosyltransferase 2 (EC 2.4.1.-) | VFG015045 | P.aeruginosa PAO1 |
| *pppA* | T6SS protein serine/threonine phosphatase PppA | VFG002061 | *P.aeruginosa* PAO1 |
| *pcr4* | Chaperone protein YscY (Yop proteins translocation protein Y) | VFG000197 | *P.aeruginosa* PAO1 |
| *flgB* | Flagellar basal-body rod protein FlgB | VFG014148 | *P.aeruginosa* PAO1 |
| *algF* | Alginate O-acetyltransferase AlgF, periplasmic | VFG000132 | *P.aeruginosa* PAO1 |
| *motC* | Flagellar motor rotation protein MotA | VFG014707 | *P.aeruginosa* PAO1 |
| *pilP* | Type IV pilus biogenesis protein PilP | VFG001219 | *P.aeruginosa* PAO1 |
| *pscJ* | Type III secretion bridge between inner and outermembrane lipoprotein (YscJ,HrcJ,EscJ, PscJ) | VFG000218 | *P.aeruginosa* PAO1 |
