# Supplementary material for: Characterization of a hemolytic and antibiotic-resistant Pseudomonas aeruginosa strain S3 pathogenic to fish isolated from Mahananda River in India
Source: PLoS One. 2024 Mar 28;19(3):e0300134. doi: 10.1371/journal.pone.0300134 (PMC10977779; doi:10.1371/journal.pone.0300134)
Supplement: Attachment — Submitted filename: Response to Reviewers.docx [file pone.0300134.s002.docx]

1. Please ensure that your manuscript meets PLOS ONE's style requirements, including those for file naming. The PLOS ONE style templates can be found at
<https://journals.plos.org/plosone/s/file?id=wjVg/PLOSOne_formatting_sample_main_body.pdf> and
<https://journals.plos.org/plosone/s/file?id=ba62/PLOSOne_formatting_sample_title_authors_affiliations.pdf>

**Author Response**- The suggested revision has been done.

.
2. Please upload a new copy of Figure 4 as the detail is not clear. Please follow the link for more information: <https://blogs.plos.org/plos/2019/06/looking-good-tips-for-creating-your-plos-figures-graphics/>" <https://blogs.plos.org/plos/2019/06/looking-good-tips-for-creating-your-plos-figures-graphics/>"

**Author Response**- A new copy of Figure 4 has been uploaded.

3. Please include captions for your Supporting Information files at the end of your manuscript, and update any in-text citations to match accordingly. Please see our Supporting Information guidelines for more information: <http://journals.plos.org/plosone/s/supporting-information>.

The suggested revision has been done.

4. When completing the data availability statement of the submission form, you indicated that you will make your data available on acceptance. We strongly recommend all authors decide on a data sharing plan before acceptance, as the process can be lengthy and hold up publication timelines. Please note that, though access restrictions are acceptable now, your entire data will need to be made freely accessible if your manuscript is accepted for publication. This policy applies to all data except where public deposition would breach compliance with the protocol approved by your research ethics board. If you are unable to adhere to our open data policy, please kindly revise your statement to explain your reasoning and we will seek the editor's input on an exemption. Please be assured that, once you have provided your new statement, the assessment of your exemption will not hold up the peer review process.

[Note: HTML markup is below. Please do not edit.]

Reviewers' comments:

Reviewer's Responses to Questions

Comments to the Author

1. Is the manuscript technically sound, and do the data support the conclusions?

The manuscript must describe a technically sound piece of scientific research with data that supports the conclusions. Experiments must have been conducted rigorously, with appropriate controls, replication, and sample sizes. The conclusions must be drawn appropriately based on the data presented.

Reviewer #1: Yes

Reviewer #2: Yes

Reviewer #3: Yes

2. Has the statistical analysis been performed appropriately and rigorously?

Reviewer #1: Yes

Reviewer #2: No

Reviewer #3: Yes

3. Have the authors made all data underlying the findings in their manuscript fully available?

The PLOS Data policy requires authors to make all data underlying the findings described in their manuscript fully available without restriction, with rare exception (please refer to the Data Availability Statement in the manuscript PDF file). The data should be provided as part of the manuscript or its supporting information, or deposited to a public repository. For example, in addition to summary statistics, the data points behind means, medians and variance measures should be available. If there are restrictions on publicly sharing data—e.g. participant privacy or use of data from a third party—those must be specified.

Reviewer #1: Yes

Reviewer #2: Yes

Reviewer #3: Yes

4. Is the manuscript presented in an intelligible fashion and written in standard English?

PLOS ONE does not copyedit accepted manuscripts, so the language in submitted articles must be clear, correct, and unambiguous. Any typographical or grammatical errors should be corrected at revision, so please note any specific errors here.

Reviewer #1: No

Reviewer #2: Yes

Reviewer #3: Yes

5. Review Comments to the Author

Please use the space provided to explain your answers to the questions above. You may also include additional comments for the author, including concerns about dual publication, research ethics, or publication ethics. (Please upload your review as an attachment if it exceeds 20,000 characters)

Reviewer #1: 1- The text of the article should be revised in terms of grammar.

**Author Response 1-** The text has been revised for grammar

2- A number of words in the pdf file are stuck together, to be checked and corrected. Words that are stuck together should be separated.

**Author Response 2-** The stuck words were separated wherever found.

3- The author claimed that the strain isolated from the river water is very similar to the standard PAO1 strain, can it be said that there is a similarity in terms of other characteristics such as the ability of hemolysis, antibiotic resistance, etc.? Explain and compare.

**Author Response 3-**

Following the suggestion, some comparisons are now included in the manuscript in the discussion section. These regions are highlighted in the manuscript.

4- Explain the reasons for conducting cytotoxicity test for the identified strain?

**Author Response 4-** Cytotoxicity test was done to comprehend the spectrum of virulence properties carried by S3.

5- What method was used to investigate the resistance of the isolated strain S3 to the colistin? Disc diffusion method?

**Author Response 5-**

Yes disc diffusion method was used to investigate the resistance of the isolated strain S3 to colistin.

However, as rightly questioned by the reviewer, three tests are accepted for colistin sensitivity test in the CLSI guidelines which includes broth micro-dilution, disk elution and colistin agar method. Thus, the disk diffusion method is not acceptable. To overcome this shortfall, we performed the disk elution method and the result showed that S3 was resistant to colistin. This information is now included in the manuscript in materials & methods section.

Reviewer #2: I have no comments

Reviewer #3: 1. It is not interesting to write S3 at the beginning of the abstract, it is better to give the first set of bacteria names (Virulent strain Pseudomonas aeruginosa) .

**Author Response 1-**The suggested revision has been done.

2. In the abstract, the names of the genes should be written in italics

**Author Response 2-**The suggested revision has been done.

3. What was the reason for not using CLSI as a reference for the antibiogram?

**Author Response 3-**We have followed both the EUCAST and CLSI guidelines but inadvertently, CLSI was not mentioned in the manuscript. This is now included.

4. "Some authors" in reference number 8 is not interesting, it is better to write "some researchers"

**Author Response 4-**The suggested revision has been done.

5. Afterincubation should be corrected in the materials and methods section (section Hemolytic activity).

**Author Response 5-**The suggested revision has been done.

6. It would have been better to determine the typing sequence of the strain using the MLST technique

**Author Response 6-**The sequence type was found as 549 using the MLST database which also matched with *P. aeruginosa* PAO1. This information is already included in the manuscript; hence no changes were done.

6. PLOS authors have the option to publish the peer review history of their article (what does this mean?). If published, this will include your full peer review and any attached files.

If you choose “no”, your identity will remain anonymous but your review may still be made public.

Do you want your identity to be public for this peer review? For information about this choice, including consent withdrawal, please see our Privacy Policy.

Reviewer #1: No

Reviewer #2: No

Reviewer #3: No

[NOTE: If reviewer comments were submitted as an attachment file, they will be attached to this email and accessible via the submission site. Please log into your account, locate the manuscript record, and check for the action link "View Attachments". If this link does not appear, there are no attachment files.]

While revising your submission, please upload your figure files to the Preflight Analysis and Conversion Engine (PACE) digital diagnostic tool, https://pacev2.apexcovantage.com/. PACE helps ensure that figures meet PLOS requirements. To use PACE, you must first register as a user. Registration is free. Then, login and navigate to the UPLOAD tab, where you will find detailed instructions on how to use the tool. If you encounter any issues or have any questions when using PACE, please email PLOS at figures@plos.org. Please note that Supporting Information files do not need this step.
